# Association between neonatal brain volume and school-age executive function in children born moderate-to-late preterm

**DOI:** 10.1038/s41390-025-04274-9

**Published:** 2025-07-24

**Authors:** Lauren Rossetti, Leona Pascoe, Rheanna M. Mainzer, Rachel Ellis, Joy E. Olsen, Deanne K. Thompson, Lex W. Doyle, Jeanie L. Y. Cheong, Peter J. Anderson

**Affiliations:** 1 School of Psychological Sciences, Turner Institute for Brain and Mental Health, Monash University, Melbourne, VIC Australia; 2 Clinical Sciences, Murdoch Children’s Research Institute, Melbourne, VIC Australia; 3 The Centre for Community Child Health, Murdoch Children’s Research Institute, Melbourne, VIC Australia; 4 Department of Paediatrics, The University of Melbourne, Melbourne, VIC Australia; 5 Clinical Epidemiology and Biostatistics Unit, Murdoch Children’s Research Institute, Melbourne, VIC Australia; 6 Newborn Research, The Royal Women’s Hospital, Melbourne, VIC Australia; 7 Developmental Imaging, Murdoch Children’s Research Institute, Melbourne, VIC Australia; 8 Department of Obstetrics, Gynaecology and Newborn Health, The University of Melbourne, Melbourne, VIC Australia; 9 Department of Pediatrics, University of California Irvine, Irvine, CA USA; 10 Center of Newborn Research, Rady Children’s Hospital of Orange County, Orange, CA, USA

## Abstract

**Background:**

Larger brain volumes in the neonatal period are associated with better 2-year cognitive development in children born moderate-to-late preterm (MLP). Whether these associations persist into school age for executive function (EF) is unknown.

**Methods:**

Children born MLP underwent brain magnetic resonance imaging (MRI) at term-equivalent age (*n* = 168) and EF assessment at 9 years (*n* = 159). Mean or median differences in EF subdomains (attentional control, cognitive flexibility, goal setting, behavioral EF) for a unit increase in brain volumes were estimated using linear regression, overall and for subgroups defined by gestational age at MRI, sex and excluding participants with developmental delay at 2 years.

**Results:**

There were few associations between brain volumes and EF. Small effects were found for larger total tissue (mean difference = 0.16; 95% CI = −0.04, 0.36; *p* = 0.11), white matter (mean difference = 0.21; 95% CI = 0.05, 0.38; *p* = 0.01) and subcortical gray matter (mean difference = 0.17; 95% CI = −0.01, 0.34; *p* = 0.06) volumes and improved goal setting. Subgroup relationships were similar.

**Conclusion:**

Neonatal brain volumes in MLP children are not strongly associated with school-age EF. Imaging techniques with higher sensitivity, and other risk factors for poorer EF should be explored.

**Impact:**

- This study described the associations between neonatal brain volumes and executive function (EF) outcomes at 9 years in children born moderate-to-late preterm (MLP), a group that has been under researched compared with children born very preterm.
- There was limited evidence of an association between neonatal brain volumes and school-age EF outcomes in children born MLP, contrasting previous findings in very preterm children.
- This suggests that neonatal brain volumes alone do not effectively predict school-age EF in children born MLP, highlighting the need for more sensitive neuroimaging techniques and identification of other important predictors of long-term outcomes in this population.

## Introduction

Preterm birth (<37 weeks’ gestation) accounts for 1 in 10 births globally and is a significant public health concern.^1^ Those born moderate-to-late preterm (MLP; 32–36 weeks’ gestation) represent 85% of all preterm births, but despite this, research on long-term outcomes for those born MLP and the factors that contribute to poorer outcomes is limited.^1,2^

Evidence increasingly suggests that children born MLP are at higher risk for poorer neurodevelopmental outcomes at school age compared with children born at term, including poorer overall cognitive function and specific cognitive skills such as executive function (EF).^3–7^ EF describes a collection of cognitive skills that support the ability to perform goal directed behavior,^8,9^ and is vital for supporting important functional outcomes, such as academic achievement and social-emotional functioning.^10–12^ Executive skills can be conceptualized within subdomains including attentional control (capacity to block out distractions and focus on one particular task), cognitive flexibility (transitioning between tasks, switching attention and effectively using working memory), goal setting (ability to reason, problem solve, plan and organize tasks strategically) and everyday executive behaviors (application of executive skills in everyday settings). Our recent meta-analysis found a pattern of EF difficulties in children born MLP at school age, compared with children born at term, specifically across the cognitive flexibility and goal setting subdomains.^5^

In the very preterm (<32 weeks’ gestational age (GA)) population, associations between neonatal brain volumes and EF outcomes at preschool and school age are well established.^13,14^ However, the same relationship cannot be assumed to exist for MLP cohorts due to the different environmental exposures experienced by MLP and very preterm infants.^15,16^ Investigating the association between neonatal brain volume and long-term EF outcomes in children born MLP could improve understanding of the biomarkers associated with future EF difficulties.

Previous work by our group,^15,17^ and others,^18^ have shown that infants born MLP at term-equivalent age have smaller brain size and regional gray and white matter volumes, larger extracerebral space, more immature gyral folding, and less-developed myelination of the posterior limb of the internal capsule, compared with infants born at term (≥37 weeks’ gestation). In addition, widespread altered white matter microstructure has been identified in infants born MLP compared with those born at term, across major fiber tracts including the corpus callosum, internal capsule, superior longitudinal fasciculus and corona radiata.^19^ Importantly, smaller total brain tissue, white matter volumes and altered white matter microstructure at term equivalent age have been associated with poorer cognitive and language outcomes at 2 years.^20,21^ Whether these brain-behavior relationships persist into school age for children born MLP is unknown. While we have recently explored neurodevelopment^6^ and EF outcomes^7^ in this population, the present study uniquely focuses on early brain volume and later EF development.

This study aimed to describe the association between MRI brain volume at term-equivalent age and EF subdomains (attentional control, cognitive flexibility, goal setting and everyday executive behaviors) at 9 years in children born MLP. We took an exploratory approach to investigate associations for total brain volume, as well as specific volumetric segments including white matter, cortical gray mater, subcortical gray matter (i.e., basal ganglia, thalamus, hippocampus, and amygdala), brainstem, cerebellum, and cerebrospinal fluid, given these associations have yet to be explored in this population. We hypothesized that smaller volumes for total brain, white matter, cortical gray matter, subcortical gray matter, brainstem and cerebellum, and larger volume for cerebrospinal fluid at term-equivalent age would be associated with poorer EF outcomes across all subdomains at 9 years.

## Methods

Infants born MLP (*n* = 201) were recruited at birth from the Royal Women’s Hospital, Melbourne, Australia, between December 2009 and November 2012 into the *“LaPrem”* longitudinal cohort study.^22,23^ Infants were excluded if they had a congenital abnormality or genetic syndrome known to affect development.

Perinatal characteristics were obtained prospectively at recruitment, through medical record review. GA was estimated using antenatal ultrasound. Six variables collected at birth were used to calculate a social risk score, including family structure, language spoken at home, education of the primary caregiver, occupation and employment status of the primary income earner, and maternal age at birth of the child.^24^ A score of 0, 1 or 2, from lowest to highest risk, was assigned to each variable and summed to generate an overall social risk score. This score was then categorized as lower (total score < 2) or higher (total score ≥ 2) social risk.

Ethics approval was provided by the Human Research Ethics Committee of the Royal Women’s Hospital (Project number: 09/38) and Royal Children’s Hospital Melbourne (HREC 35187). Written informed consent was obtained from parents for all children.

### MRI

Infants underwent MRI at term-equivalent age (between 38- and 45-weeks’ gestation) at the Royal Children’s Hospital, Melbourne. A 3 T MAGNETOM TrioTim MRI system (Siemens, Erlangen, Germany) was used, with a 12-channel circular polarized volume extremity coil. *T*_*2*_-weighted datasets were acquired with *T*_*2*_ restore turbo spin-echo sequence; voxel size, 1.0 × 1.0 × 1.0 mm (zero-filled interpolated to 0.5 × 0.5 × 1 mm); field of view, 192 × 192 mm; matrix 384 × 384; repetition time, 8910 ms; echo time, 152 ms; and flip angle, 120 degrees. Infants were scanned during natural sleep and were fed, swaddled, had earplugs and noise attenuators (MiniMuffs; Natus, Pleasanton, California) placed and were then wrapped in an infant immobilization device (MedVac; CFI Medical Solutions Inc, Fenton, Michigan). Apnea monitoring and oxygen saturation probes were used throughout the MRI, and oral sucrose was administered with parental consent, if required.

### MRI assessment

Brain volumetric segmentation was undertaken to classify the *T*_*2*_ structural MRI into white matter, cortical gray matter, subcortical gray matter (i.e., basal ganglia, thalamus, hippocampus, and amygdala), brainstem, cerebellum and cerebrospinal fluid. Morphologically adaptive neonatal tissue segmentation (MANTiS) was used to generate these volumetric segmentations, which adapts the established unified segmentation method for adult tissue classification to neonatal MRIs.^25^ For statistical analyses, brain volume variables were standardized to the mean and standard deviation (SD) of each brain region, for ease of comparability, given the significant variability in size of each volumetric segment.^26^

### Executive function assessment at 9 years

EF assessment was completed at 9 years corrected age by trained assessors unaware of participants’ GA, as part of a broader cognitive assessment battery completed across 2 days (see Cheong et al.^22^ for the *LaPrem* protocol). Assessments were completed between June 2019 and February 2024. As we have previously demonstrated a systematic lowering of scores for preterm children in cases where performance is based on chronological age, the child’s age at assessment was corrected for prematurity.^27^ EF was assessed across 4 subdomains: attentional control, cognitive flexibility, goal setting and behavioral EF. Subtest scores from the following measures were used: the Wechsler Intelligence Scale for Children, Fifth Edition, Australia and New Zealand (WISC-5; Block Design, Matrix Reasoning and Digit Span Backwards subtests),^28^ the Test of Everyday Attention for Children, Second Edition (TEA-Ch2; Hector Cancellation, Troy Dual Task, and Reds, Blues, Bags and Shoes subtests),^29^ the Contingency Naming Test (CNT),^30,31^ and the Rey Complex Figure Test (RCFT).^32,33^ These measures were categorized into EF subdomains and transformed into z-scores based on the mean and standard deviation of the *LaPrem* term control group (*n* = 134; born ≥37 weeks’ gestation, birthweight ≥2500 g). The Behavior Rating Inventory of Executive Function, Second Edition: Parent Report (BRIEF2; Global Executive Composite)^34^ was used to assess overall executive behaviors in everyday settings (behavioral EF). Table 1 details the measures and associated scores that contributed to each subdomain. Composite z-scores were computed for the attentional control, cognitive flexibility and goal setting subdomains.

**Table 1:** Executive function measure characteristics.

Executive function subdomain	Included subtests	Utilized score	Associated skill
Attentional control	Contingency Naming Test (CNT): Trial 3 (Shape Matching)^20,21^	Self-regulation score = (2 x errors) + self-corrections	Self-regulation and self-monitoring
	Contingency Naming Test (CNT): Trial 4 (Arrow Switching)^20,21^	Self-regulation score = (2 x errors) + self-corrections	Self-regulation and self-monitoring
	Test of Everyday Attention for Children, 2nd Edition (TEA-Ch2): Hector Cancellation^19^	Raw score	Selective attention
Cognitive flexibility	Wechsler Intelligence Scale for Children, 5th Edition (WISC-5): Digit Span Backwards^18^	Scaled score	Working memory
	Contingency Naming Test (CNT): Trial 3 (Shape Matching)^20,21^	Efficiency score = [(1/time) / √(errors + 1)] × 100	Shifting
	Contingency Naming Test (CNT)^20,21^: Trial 4 (Arrow Switching)^20,21^	Efficiency score = [(1/time) / √(errors + 1)] x 100	Shifting
	Test of Everyday Attention for Children, 2nd Edition (TEA-Ch2): Reds and Blues, Bags and Shoes^19^	Raw score	Shifting
	Test of Everyday Attention for Children, 2nd Edition (TEA-Ch2): Troy Dual Task^19^	Raw score	Divided attention
Goal setting	Wechsler Intelligence Scale for Children, 5th Edition (WISC-5): Block Design^18^	Scaled score	Visual spatial reasoning and problem solving
	Wechsler Intelligence Scale for Children, 5th Edition (WISC-5): Matrix Reasoning^18^	Scaled score	Visual spatial reasoning
	Rey Complex Figure Test (RCFT)^22,23^	Accuracy score	Organization and planning
		Organizational Strategy score	Organization and planning
Behavioral executive function	Behavior Rating Inventory of Executive Function, Second Edition (BRIEF2): Parent Report^24^	Global Executive Composite (GEC) T-score	Global behavioral executive function

### Statistical analyses

Stata 18 was used for data analyses.^35^ Participant characteristics were compared between children with and without both neonatal brain volume and 9-year EF data using descriptive statistics. Differences in mean or median EF subdomains at 9 years for a standard deviation increase in neonatal MRI volumes were estimated using linear regression (for symmetrically distributed outcomes) or quantile regression (for skewed outcomes). Separate models were fitted for each volume and EF subdomain score. To account for clustering of siblings from multiple births, linear regression models were fitted using generalized estimating equations and robust standard errors. Cluster robust standard errors were used for quantile regression models.

Subgroup analyses were performed in order to describe how the associations between neonatal brain volumes and EF subdomains at 9 years differed according to participants’ GA at the time of the MRI and sex (male and female), as there is evidence that these factors influence neonatal brain volume.^36^ We defined three subgroups for GA at MRI based on the similar brain volumes expected during these gestational weeks:^36,37^ Group 1 (38 to 39 weeks’ GA), Group 2 (40 to 41 weeks’ GA), and Group 3 (≥42 weeks’ GA). An additional subgroup analysis was performed excluding participants with developmental delay at 2 years (defined as any cerebral palsy or any score <−1 SD below the mean for children born at term on either cognitive, language, and/or motor development on the Bayley Scales of Infant and Toddler Development, 3rd Edition).

To account for missing EF data at 9 years, multiple imputation by chained equations was used. The imputation models included the neonatal brain volume metric, EF outcome, sex, multiple birth, social risk, and developmental delay at 2 years, and a separate multiple imputation procedure was performed for each predictor-outcome combination and each subgroup. Linear and logistic regression was used to impute continuous, normally distributed and dichotomous variables, respectively. Predictive mean matching was used to impute skewed continuous outcome variables. Forty imputations were performed, with Rubin rules used to obtain estimates and standard errors.^38^ A missingness-directed acyclic graph, presented in Supplementary Fig. S1, details the justification for using multiple imputation and our assumptions regarding the causes of missing data. For comparison, complete case analyses were also performed.

## Results

One hundred and ninety-nine (99%) of the 201 MLP infants who were recruited at birth underwent MRI at term-equivalent age. Volumetric segmentation was performed on 168 (84%) scans, with motion artefact the primary reason for missing volumetric segmentation.^39^ 159 (79%) children completed EF assessment at 9 years of age; 37 of the original cohort were non-contactable or declined, and 5 withdrew participation. Of the 168 participants with neonatal brain volumetric data, 135 (67%) completed EF assessment at 9 years.

Participant characteristics are presented in Table 2 and MRI characteristics are presented in Table 3. As expected for children born preterm, participants had high rates of antenatal corticosteroid use, multiple birth, and cesarean birth. A large proportion (44%) of participants had developmental delay at 2 years of age. Characteristics between children with neonatal brain volume data and EF data at 9 years, versus those without these data, were largely comparable (see Supplementary Tables S1 and S2). MLP children without neonatal brain volume or 9-year EF data were more likely to have been born via assisted conception, their mothers to have received antenatal magnesium sulfate, and to be male, have slightly higher birth weight, and higher rates of developmental delay at 2 years than those with data. Those with data had mothers who were slightly older and were more likely to be of multiple birth and cesarean birth. MRI characteristics by sex and GA subgroups are summarized in Table 4.

**Table 2:** Participant characteristics for MLP children with neonatal MRI brain volume and 9-year EF data.

Variable	MLP (*n* = 135)
*Perinatal characteristics*	
Maternal age (years), mean (SD)	34.2 (4.5)
Maternal preeclampsia	24 (18%)
Assisted conception *n* = 133	24 (18%)
Antenatal corticosteroid use	81 (60%)
Antenatal magnesium sulfate	7 (5%)
Multiple birth	51 (38%)
Cesarean birth	93 (69%)
*Neonatal characteristics*	
Male sex	58 (43%)
Gestational age at birth (weeks), mean (SD)	34.3 (1.2)
Birth weight (g), mean (SD)	2142 (426)
Birth weight z-score, mean (SD)	−0.3 (0.9)
Apgar score @ 5 min, median (IQR)	9 (8,9)
Any respiratory support	18 (13%)
Neonatal hospitalization (days), median (IQR) *n* = 130	19.5 (14,29)
Higher social risk (neonatal period) *n* = 134	45 (34%)
Developmental delay at 2 years^a^	59 (44%)
*9-year assessment*	
Age at assessment (years), mean (SD)	9.7 (0.4)
Higher social risk at 9 years, *n* = 134	34 (25%)
Attentional control composite score (z-score) *n* = 122	−0.01 (0.73)
Cognitive flexibility composite score (z-score) *n* = 117	−0.05 (0.64)
Goal setting composite score (z-score) *n* = 103	−0.26 (0.78)
Behavioral EF score (BRIEF2 GEC T-score), median (IQR) *n* = 131	51 (45, 61)

Statistics are number (%), unless otherwise stated.

*BRIEF2* Behavior Rating Inventory of Executive Function Second Edition, *EF* executive function, *GEC* Global Executive Composite, *IQR* interquartile range, *MLP* moderate-to-late preterm, *SD* standard deviation.

^a^Developmental delay at 2 years defined as any cerebral palsy or any delay on either cognitive, language, and/or motor development from the Bayley-III.

**Table 3:** Neonatal MRI characteristics and brain volumes for participants with neonatal brain volume data and EF data at 9 years.

Variable	MLP *n* = 135
Neonatal characteristics at MRI	
Gestational age at MRI corrected (weeks)	41.4 (1.2)
Weight (g)	3312 (538)
Head circumference (cm)	35.8 (1.5)
Volume	**Raw measure (cc)**
Total brain tissue	401.8 (37.8)
White matter	158.5 (14.6)
Cortical gray matter	177.4 (18.3)
Subcortical gray matter	31.7 (2.8)
Cerebellum	27.7 (3.2)
Brainstem	6.4 (0.6)
Cerebrospinal fluid	91.6 (16.8)

Statistics are mean (SD). The standardized measures were standardized to the mean and standard deviation (SD) of each brain region, given the significant variability in size of each volumetric segment.^23^

*cc* cubic centimeters, *MLP* moderate-to-late preterm, *MRI* magnetic resonance imaging, *SD* standard deviation.

**Table 4:** Neonatal MRI characteristics and brain volumes by subgroups for corrected gestation age at time of MRI and sex, for participants with neonatal brain volume and EF data at 9 years.

	GA at time of MRI Subgroup			Sex Subgroup	
Variable	38–39 weeks (*n* = 14)	40–41 weeks (*n* = 73)	≥42 weeks (*n* = 48)	Males (*n* = 58)	Females (*n* = 77)
*Neonatal characteristics at MRI*					
GA corrected at MRI (weeks)	39.1 (0.5)	41.1 (0.6)	42.6 (0.6)	41.3 (1.3)	41.4 (1.1)
GA corrected at MRI (weeks) range (min, max)	38.4, 39.9	40, 41.9	42, 44.1	38.4, 43.9	38.4, 44.1
Weight (g)	2659 (302)	3292 (405)	3534 (609)	3350 (642)	3283 (446)
Head circumference (cm)	34.2 (0.8)	35.8 (1.1)	36.3 (1.8)	35.9 (1.5)	35.8 (1.4)
*Volume (cc)*					
Total brain tissue	353.2 (39.2)	398.9 (26.7)	420.4 (38.6)	408.5 (41.0)	396.8 (34.6)
White matter	141.3 (15.9)	157.9 (10.6)	164.5 (15.5)	161.4 (15.7)	156.4 (13.5)
Cortical gray matter	154.8 (19.2)	175.6 (13.2)	186.7 (18.5)	180.5 (20.4)	175.1 (16.4)
Subcortical gray matter	28.6 (2.8)	31.5 (2.2)	32.9 (2.8)	32.1 (2.9)	31.4 (2.7)
Cerebellum	22.9 (2.1)	27.5 (2.2)	29.6 (3.1)	28.0 (3.4)	27.6 (3.0)
Brainstem	5.6 (0.3)	6.4 (0.5)	6.7 (0.6)	6.5 (0.7)	6.4 (0.6)
Cerebrospinal fluid	75.9 (7.9)	89.9 (15.0)	98.8 (17.7)	95.4 (20.1)	88.7 (13.2)

Data are mean (SD) unless otherwise specified. n = 96 for weight and head circumference for GA Group 2.

*cc* cubic centimeters, *GA* gestational age, *MRI* magnetic resonance imaging, *SD* standard deviation.

### Association between brain volumes and 9-year executive function outcomes

**Fig. 1:**
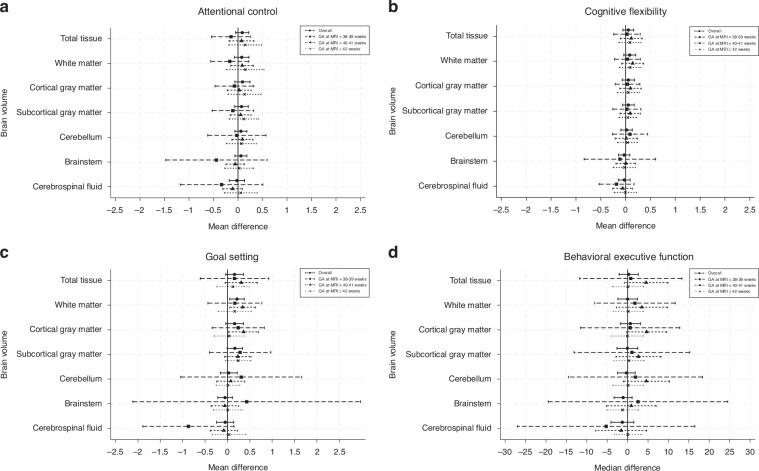
Mean or median differences in executive function variables at 9 years for a 1 standard deviation increase in neonatal brain volumes for children born moderate-to-late preterm, overall and by gestational age at MRI subgroup. Points represent the effect size and lines represent the 95% confidence intervals. For (**a**), (**b**) and (**c**), the points represent the mean difference in cognitive score associated with a 1 SD increase in brain volume. For (**d**), the points represent the median difference in Behavior Rating Inventory of Executive Function – Second Edition – Global Executive Composite (BRIEF2 GEC) T-Score associated with a 1 SD increase in brain volume. Points to the right of the line indicate larger brain volume is associated with better performance. Values have been reverse-scored in (**d**), so that points to the right of the line indicate larger brain volume is associated with less parent-reported behavioral executive function difficulties.

The associations between neonatal brain volumes and 9-year EF subdomains, both overall and by subgroups of corrected GA at time of MRI are presented in Fig. 1. Overall, effect sizes were small, with little evidence of an association between neonatal brain volumes and 9-year EF subdomains. While small, the most considerable effect sizes overall, were found for larger total tissue (mean difference = 0.16; 95% CI = −0.04, 0.36; *p* = 0.11), white matter (mean difference = 0.21; 95% CI = 0.05, 0.38; *p* = 0.01), cortical gray matter (mean difference = 0.16; 95% CI = −0.04, 0.36; *p* = 0.11) and subcortical gray matter (mean difference = 0.17; 95% CI = −0.01, 0.34; *p* = 0.06) volumes and improved goal setting performance.

**Fig. 2:**
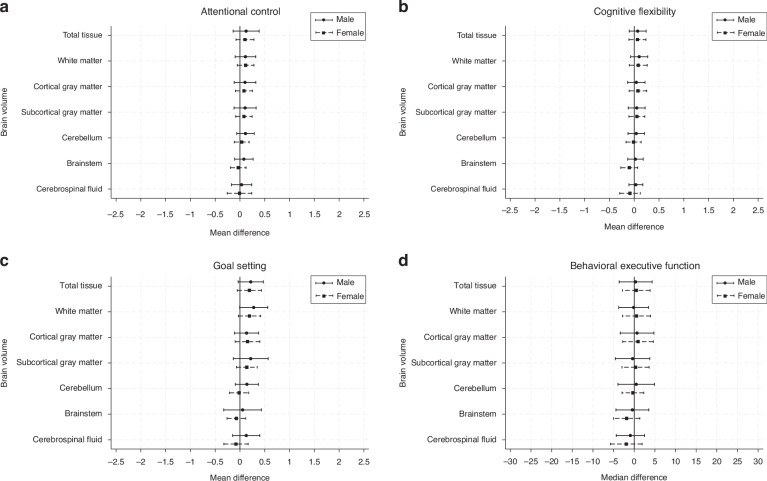
Mean or median differences in executive function variables at 9 years for a 1 standard deviation increase in neonatal brain volumes for children born moderate-to-late preterm by sex subgroup. Points represent the effect size and lines represent the 95% confidence intervals. For (**a**), (**b**) and (**c**), the points represent the mean difference in cognitive score associated with a 1 SD increase in brain volume. For (**d**), the points represent the median difference in Behavior Rating Inventory of Executive Function – Second Edition – Global Executive Composite (BRIEF2 GEC) T-Score associated with a 1 SD increase in brain volume. Points to the right of the line indicate larger brain volume is associated with better performance. Values have been reverse-scored in (**d**), so that points to the right of the line indicate larger brain volume is associated with less parent-reported behavioral executive function difficulties.
- Excluding participants with developmental delay
  The subgroup analysis excluding participants with developmental delay at 2 years was largely comparable to the total sample estimates, showing limited evidence of associations (Supplementary Table S7). There was a slight weakening of the strength of associations for the cognitive flexibility, goal setting and behavioral EF subdomains and a small strengthening of associations for the attentional control subdomain.

Subgroup analyses:

- GA at MRI
  Consistent with the overall findings, effect sizes were small for subgroups based on GA at MRI. Whilst similar effect sizes were found for the associations between total tissue, white matter, cortical gray matter and subcortical gray matter volumes and goal setting, there was less precision in these estimates for the subgroups based on GA at MRI. For the attentional control subdomain, a trend was observed in that later GA at MRI was associated with a larger effect size for total tissue, white matter, and cortical and subcortical gray matter volumes.
- Sex
  For sex, again effect sizes were small between the brain volumes and EF subdomains for the male and female subgroups (see Fig. 2). The estimates obtained from the complete case analyses (Supplementary Tables S3–S6) were comparable to the estimates obtained from multiple imputation.

## Discussion

In general, we found little evidence of associations between neonatal brain volumes and EF outcomes at 9 years. This pattern of limited associations was observed for both cognitive composite scores (attentional control, cognitive flexibility and goal setting) and the parent-reported outcome (behavioral EF). The most notable associations were seen for larger total tissue, white matter, cortical gray matter and subcortical gray matter volumes and improved goal setting performance, though these effects were small and unlikely to be clinically important. Given the range in GA at MRI (38.4 to 45 weeks) and the established increase in brain volume that occurs daily following birth,^36^ subgroup analyses were performed to explore whether associations were consistent for those scanned at slightly different timepoints. Effect sizes for the associations between neonatal brain volumes and school-age EF outcomes across these GA subgroups were also small. There was similarly little evidence of associations for subgroups by sex and when excluding participants with developmental delay at 2 years.

Previous literature exploring associations between neonatal MRI metrics and school-age cognitive outcomes for preterm children is mostly reported in the very and extremely preterm cohorts.^13,14,40–44^ For EF specifically, associations have been found between larger neonatal brain volumes (including total brain tissue, thalamic, cerebellum, and cortical gray matter) and improved goal setting and fewer parent-reported EF behavioral challenges at preschool age and early adolescence in those born preterm.^13,45^ Associations between white matter abnormalities and working memory performance in children born very preterm have also been found at 2 and 4 years of age.^14,40^ While we found limited evidence of an association, the consistent pattern of positive coefficients between neonatal total brain tissue, cortical and subcortical gray matter, and white matter volumes with goal setting outcomes, reflects a similar but weaker pattern of findings to those in children born very preterm.^13^ These findings may reflect less severe brain alterations seen at term-equivalent age and more subtle EF difficulties at school age for those born MLP compared with preterm peers born at earlier GAs.^5,15^

Our findings at 9 years of age may be considered in contrast to our previously reported findings at 2 years, where positive associations were identified between total brain tissue, white matter and cerebellum volumes at term-equivalent age and cognitive scores at 2 years old in the same cohort of children born MLP.^21^ Our results indicate that the relationship between neonatal brain volumes and cognitive outcomes may not extend into school age for children born MLP, despite associations observed at approximately 2 years for both very preterm and MLP cohorts.^21,42,46–50^ Neonatal brain volumes may have greater sensitivity in predicting short-term, rather than long-term neurodevelopmental outcomes in the MLP population.^21,42,51^ Other environmental factors, such as social risk, possibly play a more prominent role in the development and progression of cognitive skills through later childhood, particularly for children born MLP who have more subtle brain alterations compared with those born at earlier gestations.^42^ Other environmental influences such as parenting style,^52^ and access to educational opportunities influenced by socioeconomic status,^53,54^ may significantly affect EF development during the years between infancy and middle childhood, potentially attenuating the predictive utility of early biological factors. A multimodal modeling approach combining imaging and clinical data may also enhance outcome predictions in this population.^55^

The neural foundations of EF are complex, with protracted and dynamic development of these brain networks across childhood.^56,57^ Developmental plasticity may attenuate the predictive utility of early brain volume data, given children may establish alternative neural pathways that compensate for structural differences observed in the neonatal period.^58^ Intricate network connections across major white matter tracts play important roles in the execution and development of these skills.^59–61^ Given this, volumetric measurements alone may not adequately capture the elaborate neural substrates underlying EF skills, and measures of neonatal white matter integrity may be more sensitive markers of later cognitive function. Abnormalities in white-matter microstructure across important tracts have been found in infants born MLP,^19^ which suggests a future avenue to explore the predictive utility of these MRI metrics on long-term executive outcomes in this population.

### Strengths and limitations

The current study utilized a prospective, longitudinal design and a well-characterized MLP cohort. We undertook a comprehensive assessment of EF, which allowed us to explore executive performance across specific subdomains, based on the aggregation of multiple measures that targeted skills within each of these areas. It is important to note that this sample of MLP children may not be representative of the broader MLP population. Our participants were recruited from a single tertiary maternity hospital and may have included a higher-than-expected proportion of more vulnerable infants, who may be more likely to have more morbidities including poor neurodevelopment. Importantly, sensitivity analyses excluding children with developmental delay at 2 years did not substantially alter conclusions from this study. The follow-up rate of our cohort at 9-years was affected by COVID-19 restrictions, and as a result is lower than anticipated. Selection bias was, however, addressed using multiple imputation. The current study may also be underpowered to detect associations, particularly the subtle associations expected in MLP populations, given that the sample size is pre-existing, rather than being powered specifically for this research question.

## Conclusions

Our findings revealed little evidence of an association between brain volumes at term-equivalent age and EF outcomes at 9-years in children born MLP. We explored associations by subgroups for sex and GA at MRI and found a similar pattern of evidence. While early MRI brain volumes may prove useful in predicting outcomes at 2 years of age for children born MLP, they may not be the most sensitive neural marker of school-age executive outcomes. The complex developmental processes and environmental interactions that shape EF networks in the intervening years likely have a significant impact on the predictive validity of brain volume on later outcomes for this population. It is important to acknowledge that methodological constraints, including the small sample size and heterogeneity within the MLP cohort may have contributed to the lack of associations found in this study. Further research should explore other imaging modalities and their utility in predicting long-term EF outcomes in children born MLP, such as diffusion-weighted imaging and functional connectivity analysis. Postnatal environmental factors that may influence EF outcomes should also be examined. The current study highlights the complexity of brain-behavior relationships and the need for continued research to better understand the unique profile of risk for children born MLP who experience later neurodevelopmental difficulties, including in EF.

## Supplementary information

Supplementary Material

## Data Availability

The datasets generated during and/or analysed during the current study are available from the corresponding author on reasonable request.
