# Supplementary material for: Association between neonatal brain volume and school-age executive function in children born moderate-to-late preterm
Source: Pediatr Res. 2025 Jul 24;99(3):1060–7. doi: 10.1038/s41390-025-04274-9 (PMC13021516; doi:10.1038/s41390-025-04274-9)
Supplement: Supplementary file 1 — Supplementary Material [file 41390_2025_4274_MOESM1_ESM.pdf]

Association Between Neonatal Brain Volume and School-Age Executive Function in Children Born Moderate-to-Late Preterm

SUPPLEMENTARY MATERIAL

Lauren Rossetti[a,b], Leona Pascoe[a,c,d], Rheanna M. Mainzer[d,e], Rachel Ellis[b], Joy E. Olsen[b,f], Deanne K. Thompson[a,b,d,g], Lex W. Doyle[b,f,h], Jeanie L. Y. Cheong[b,d,f,h*] and Peter J. Anderson[a,b,i,j*]

Affiliations:

a. School of Psychological Sciences, Turner Institute for Brain and Mental Health, Monash University, Melbourne, Australia
b. Clinical Sciences, Murdoch Children's Research Institute, Melbourne, Australia
c. The Centre for Community Child Health, Murdoch Children's Research Institute, Melbourne, Australia
d. Department of Paediatrics, The University of Melbourne, Melbourne, Australia
e. Clinical Epidemiology and Biostatistics Unit, Murdoch Children's Research Institute, Melbourne, Australia
f. Newborn Research, The Royal Women's Hospital, Melbourne, Australia
g. Developmental Imaging, Murdoch Children's Research Institute, Melbourne, Australia
h. Department of Obstetrics, Gynaecology and Newborn Health, The University of Melbourne, Melbourne, Australia
i. Department of Pediatrics, University of California Irvine, California
j. Centre of Newborn Research, Children's Hospital of Orange County, Orange, California

**** Contributed equally as co-senior authors.***

Address correspondence to: Lauren Rossetti, School of Psychological Sciences, Turner Institute for Brain and Mental Health, 18 Innovation Walk, Monash University, Clayton, VIC, 3800, AUS. Email: laurenrossetti1@gmail.com

Supplementary Table S1. Neonatal MRI characteristics and brain volumes for participants with neonatal brain volume and EF data at 9 years, vs those without neonatal brain volume and/or EF data.

Variable	MLP group (n = 201)	
	**With data n=135**	**Without data n=66**
**Perinatal characteristics**		
Maternal age (years), mean (SD)	34.2 (4.5)	33.6 (5.2)
Maternal preeclampsia	24 (18%)	12 (18%)
Assisted conception	24 (18%) n=133	15 (23%) n=65
Antenatal corticosteroid use	81 (60%)	40 (61%)
Antenatal magnesium sulfate	7 (5%)	8 (12%)
Multiple birth	51 (38%)	23 (35%)
Cesarean birth	93 (69%)	44 (67%)
**Neonatal characteristics**		
Male sex	58 (43%)	40 (61%)
Gestational age at birth (weeks), mean (SD)	34.3 (1.2)	34.7 (1.2)
Birth weight (g), mean (SD)	2142 (426)	2200 (531)
Birth weight z-score, mean (SD)	-0.3 (0.9)	-0.4 (1.2)
Apgar score @ 5 minutes, median (IQR)	9 (8,9)	9 (8,9)
Any respiratory support	18 (13%)	10 (15%)
Neonatal hospitalization (days), median (IQR)	19.5 (14,29) n=130	20 (13, 25) n=63
Higher social risk (neonatal period)	45 (34%) n=134	21 (34%) n=62
Developmental delay at 2 years#	59 (44%)	43 (68%) n=63

Statistics are number (%) unless stated otherwise. #Developmental delay at 2 years defined as any cerebral palsy or any developmental delay on either cognitive, language, and/or motor development.

Abbreviations: EF = executive function; IQR = interquartile range; MLP = moderate-to-late preterm; SD = standard deviation.

Supplementary Table S2. Demographic characteristics and MRI variables for participants with and without EF data at 9 years.

Variable	MLP group	
	**With EF data n=135**	**Without data n=33**
**Characteristics at MRI**		
Gestational age corrected (weeks), mean (SD)	41.4 (1.2)	41.6 (1.0) n=31
Weight (g), mean (SD)	3312 (538)	3533 (624) n=29
Head circumference (cm), mean (SD)	35.8 (1.5)	36.2 (1.2) n=29
**MRI volume variables**		
Total brain tissue volume (cc), mean (SD)	401.8 (37.8)	398.7 (49.6)
White matter volume (cc), mean (SD)	158.5 (14.6)	156.0 (20.0)
Cortical gray matter volume (cc), mean (SD)	177.4 (18.3)	177.6 (24.0)
Subcortical gray matter volume (cc), mean (SD)	31.7 (2.8)	31.5 (3.7)
Cerebellum (cc), mean (SD)	27.7 (3.2)	27.2 (3.1)
Brainstem (cc), mean (SD)	6.4 (0.6)	6.3 (0.6)
Cerebrospinal fluid volume (cc), mean (SD)	91.6 (16.8)	92.5 (20.0)

Statistics are number (%) unless stated otherwise. Abbreviations: cc = cubic centimeters; EF = executive function; MLP = moderate-to-late preterm; MRI = magnetic resonance imaging; SD = standard deviation.

Supplementary Table S3. Mean difference in attentional control for a 1 standard deviation increase in brain volume for children born moderate-to-late preterm. Estimates are obtained from an analysis of complete cases.

	Overall	GA at time of MRI			Sex	
**MRI Variable**	**(n=122)**	**38-39 weeks (n=13)**	**40-41 weeks (n=67)**	**≥42 weeks (n=42)**	**Males (n=48)**	**Females (n=74)**
Total brain tissue	0.07 (-0.04, 0.18) p=.21	-0.06 (-0.35, 0.22) p=.67	0.09 (-0.11, 0.29) p=.39	0.22 (-0.04, 0.47) p=.09*	0.13 (-0.02, 0.29) p=.10	0.08 (-0.07, 0.22) p=.30*
White matter	0.07 (-0.05, 0.19) p=.27	-0.07 (-0.37, 0.23) p=.65	0.08 (-0.10, 0.26) p=.39	0.23 (-0.04, 0.51) p=.09*	0.14 (-0.03, 0.31) p=.10	0.09 (-0.06, 0.23) p=.24*
Cortical gray matter	0.08 (-0.03, 0.18) p=.16	-0.06 (-0.32, 0.21) p=.66	0.08 (-0.13, 0.30) p=.44	0.21 (-0.02, 0.44) p=.07*	0.12 (-0.03, 0.27) p=.13	0.07 (-0.09, 0.23) p=.37*
Subcortical gray matter	0.06 (-0.04, 0.15) p=.24	-0.09 (-0.38, 0.20) p=.56	0.10 (-0.07, 0.26) p=.26	0.19 (-0.07, 0.45) p=.15*	0.12 (-0.03, 0.26) p=.11	0.09 (-0.05, 0.22) p=.21*
Cerebellum	0.05 (-0.06, 0.15) p=.40	0.06 (-0.23, 0.34) p=.70	0.08 (-0.13, 0.29) p=.44	0.12 (-0.12, 0.35) p=.32*	0.11 (-0.04, 0.26) p=.15	0.03 (-0.10, 0.17) p=.61*
Brainstem	-0.01 (-0.11, 0.09) p=.85	-0.26 (-0.86, 0.34) p=.40	-0.02 (-0.19, 0.16) p=.84	0.08 (-0.15, 0.32) p=.49*	0.08 (-0.06, 0.22) p=.26	-0.04 (-0.16, 0.09) p=.57*
Cerebrospinal fluid	-0.03 (-0.17, 0.11) p=.70	-0.20 (-0.52, 0.13) p=.24	-0.12 (-0.38, 0.14) p=.37	0.14 (-0.28, 0.56) p=.50*	0.03 (-0.13, 0.20) p=.68	-0.03 (-0.31, 0.24) p=.80*

*Estimated linear regression with cluster robust standard errors for multiple births due to failure to fit models using GEEs

Abbreviations: GA = gestational age; MRI = magnetic resonance imaging

Supplementary Table S4. Mean difference in cognitive flexibility for a 1 standard deviation increase in brain volume for children born moderate-to-late preterm. Estimates are obtained from an analysis of complete cases.

	**Overall**	**GA at time of MRI**			**Sex**	
**MRI Variable**	**(n=117)**	**38-39 weeks (n=13)**	**40-41 weeks (n=62)**	**≥42 weeks (n=42)**	**Males (n=45)**	**Females (n=72)**
Total brain tissue	0.07 (-0.06, 0.20) p=.27	-0.02 (-0.25, 0.20) p=.83	0.13 (-0.10, 0.36) p=.27	0.08 (-0.15, 0.31) p=.49	0.06 (-0.12, 0.24) p=.50	0.06 (-0.11, 0.24) p=.47
White matter	0.09 (-0.04, 0.22) p=.18	-0.03 (-0.26, 0.19) p=.77	0.14 (-0.09, 0.37) p=.24	0.11 (-0.11, 0.33) p=.33	0.09 (-0.08, 0.27) p=.30	0.07 (-0.10, 0.25) p=.42
Cortical gray matter	0.07 (-0.06, 0.20) p=.27	-0.02 (-0.24, 0.20) p=.86	0.14 (-0.08, 0.36) p=.21	0.07 (-0.17, 0.31) p=.58	0.05 (-0.13, 0.23) p=.58	0.08 (-0.10, 0.25) p=.40
Subcortical gray matter	0.05 (-0.07, 0.18) p=.38	-0.05 (-0.29, 0.19) p=.67	0.09 (-0.15, 0.32) p=.47	0.07 (-0.13, 0.26) p=.50	0.03 (-0.15, 0.21) p=.76	0.06 (-0.09, 0.21) p=.45
Cerebellum	0 (-0.11, 0.11) p=.99	0.06 (-0.24, 0.36) p=.69	-0.03 (-0.26, 0.20) p=.80	0.01 (-0.18, 0.19) p=.96	0.01 (-0.15, 0.17) p=.90	-0.02 (-0.17, 0.13) p=.77
Brainstem	-0.04 (-0.14, 0.06) p=.40	-0.29 (-0.88, 0.31) p=.35	-0.03 (-0.21, 0.15) p=.74	-0.16 (-0.36, 0.04) p=.11	0.01 (-0.13, 0.14) p=.89	-0.09 (-0.24, 0.06) p=.24
Cerebrospinal fluid	-0.03 (-0.16, 0.10) p=.64	-0.26 (-0.49, -0.03) p=.03	-0.07 (-0.31, 0.17) p=.56	-0.01 (-0.21, 0.19) p=.93	0.03 (-0.13, 0.19) p=.75	-0.08 (-0.34, 0.18) p=.54

*Estimated linear regression with cluster robust standard errors for multiple births due to failure to fit models using GEEs
Abbreviations: GA = gestational age; MRI = magnetic resonance imaging

Supplementary Table S5. Mean difference in goal setting for a 1 standard deviation increase in brain volume for children born moderate-to-late preterm. Estimates are obtained from an analysis of complete cases.

	**Overall**	**GA at time of MRI**			**Sex**	
**MRI Variable**	**(n=103)**	**38-39 weeks (n=11)**	**40-41 weeks (n=54)**	**≥42 weeks (n=38)**	**Males (n=38)**	**Females (n=65)**
Total brain tissue	0.15 (-0.05, 0.35) p=.14	0.28 (-0.26, 0.82) p=.25*	0.30 (-0.04, 0.64) p=.08	0.11 (-0.22, 0.45) p=.51	0.12 (-0.16, 0.40) p=.41	0.15 (-0.09, 0.40) p=.22
White matter	0.21 (0.00, 0.41) p=.05	0.24 (-0.28, 0.77) p=.30*	0.30 (-0.01, 0.62) p=.06	0.20 (-0.16, 0.57) p=.27	0.17 (-0.14, 0.49) p=.28*	0.18 (-0.06, 0.43) p=.14
Cortical gray matter	0.14 (-0.06, 0.34) p=.18	0.29 (-0.22, 0.80) p=.21*	0.33 (-0.02, 0.67) p=.07	0.06 (-0.26, 0.39) p=.70	0.07 (-0.20, 0.34) p=.63	0.17 (-0.10, 0.43) p=.21
Subcortical gray matter	0.15 (-0.04, 0.33) p=.12	0.17 (-0.47, 0.82) p=.60	0.22 (-0.04, 0.49) p=.10	0.16 (-0.17, 0.50) p=.34	0.13 (-0.17, 0.42) p=.40	0.15 (-0.07, 0.37) p=.18
Cerebellum	-0.02 (-0.19, 0.14) p=.77	0.43 (-0.11, 0.96) p=.12	0.05 (-0.31, 0.40) p=.79	-0.07 (-0.31, 0.17) p=.58	-0.03 (-0.34, 0.29) p=.87	-0.03 (-0.24, 0.17) p=.75
Brainstem	-0.09 (-0.24, 0.07) p=.27	-0.54 (-1.67, 0.59) p=.35	-0.10 (-0.36, 0.16) p=.45	-0.04 (-0.30, 0.23) p=.79	-0.08 (-0.43, 0.28) p=.68	-0.08 (-0.25, 0.10) p=.39
Cerebrospinal fluid	-0.07 (-0.26, 0.13) p=.51	-1.00 (-1.20, -0.79) p<.001	-0.08 (-0.40, 0.23) p=.61	0.08 (-0.26, 0.43) p=.65	-0.05 (-0.34, 0.24) p=.75	-0.08 (-0.34, 0.18) p=.54

*Estimated linear regression with cluster robust standard errors for multiple births due to failure to fit models using GEEs.
Abbreviations: GA = gestational age; MRI = magnetic resonance imaging

Supplementary Table S6. Median difference in behavioral executive function for a 1 standard deviation increase in brain volume for children born moderate-to-late preterm. Estimates are obtained from an analysis of complete cases.

	Overall Sample	GA at time of MRI Subgroup			Sex Subgroup	
**MRI Variable**	**(n=131)**	**38-39 weeks (n=12)**	**40-41 weeks (n=72)**	**≥42 weeks (n=47)**	**Males (n=55)**	**Females (n=76)**
Total brain tissue	0 (-2.94, 2.94) p=1.00	0 (-20.12, 20.12) p=1.00	-4.72 (-10.08, 0.64) p=.08	-0.54 (-5.39, 4.30) p=.82	-0.60 (-6.59, 5.39) p=.84	-1.04 (-4.82, 2.74) p=.59
White matter	0 (-3.00, 3.00) p=1.00	0 (-14.62, 14.62) p=1.00	-4.59 (-9.26, 0.08) p=.05	-0.44 (-5.38, 4.49) p=.86	-0.50 (-6.33, 5.34) p=.86	-0.77 (-4.24, 2.70) p=.66
Cortical gray matter	-0.67 (-3.50, 2.17) p=.64	0 (-24.88, 24.88) p=1.00	-3.88 (-8.93, 1.18) p=.13	-0.65 (-6.02, 4.71) p=.81	-0.68 (-6.52, 5.15) p=.82	-1.13 (-5.07, 2.81) p=.57
Subcortical gray matter	0 (-3.16, 3.16) p=1.00	-3.17 (-13.73, 7.39) p=.52	-3.74 (-8.88, 1.39) p=.15	-0.72 (-5.28, 3.84) p=75	-0.47 (-6.84, 5.89) p=.88	-0.99 (-4.40, 2.43) p=.57
Cerebellum	0 (-2.67, 2.67) p=1.00	-1.92 (-22.23, 18.40) p=.84	-3.63 (-9.17, 1.91) p=.20	-0.30 (-4.80, 4.20) p=.89	-2.15 (-7.90, 3.61) p=.46	0.52 (-1.81, 2.85) p=.66
Brainstem	1.30 (-1.40, 4.00) p=.34	0 (-26.86, 26.86) p=1.00	-3.32 (-8.18, 1.54) p=.18	0.84 (-3.46, 5.13) p=.70	0.52 (-5.55, 6.58) p=.87	1.34 (-1.47, 4.15) p=.35
Cerebrospinal fluid	1.40 (-1.01, 3.80) p=.25	7.01 (-20.38, 34.40) p=.58	0 (-4.64, 4.64) p=1.00	-0.39 (-4.46, 3.69) p=.85	0.45 (-3.41, 4.31) p=.82	1.79 (-1.57, 5.14) p=.29

Estimated using quantile regression due to skewed continuous outcome variable

Abbreviations: GA = gestational age; MRI = magnetic resonance imaging

Supplementary Table S7. Mean difference in EF composite scores for a 1 standard deviation increase in brain volume for children born moderate-to-late preterm, excluding participants with developmental delay at 2 years. Estimates are obtained from an analysis of complete cases.

MRI Variable	Attentional Control (n=70)	Cognitive Flexibility (n=66)	Goal Setting (n=60)	Behavioural EF (n=75)
Total brain tissue	0.11 (-0.02, 0.24) p=.10	0.01 (-0.13, 0.16) p=.88	-0.02 (-0.23, 0.20) p=.86	0.93 (-1.86, 3.72) p=.51
White matter	0.10 (-0.04, 0.23) p=.17	0 (-0.14, 0.14) p=.96	-0.01 (-0.22, 0.20) p=.94	0.83 (-1.95, 3.61) p=.55
Cortical gray matter	0.12 (-0.01, 0.25) p=.07	0.03 (-0.12, 0.18) p=.70	-0.02 (-0.23, 0.20) p=.89	1.00 (-1.73, 3.73) p=.47
Subcortical gray matter	0.12 (-0.01, 0.25) p=.08	0.01 (-0.14, 0.15) p=.93	0.02 (-0.20, 0.23) p=.89	0.97 (-1.94, 3.87) p=.51
Cerebellum	0.09 (-0.04, 0.22) p=.18	-0.02 (-0.16, 0.12) p=.77	-0.08 (-0.28, 0.12) p=.43	0.82 (-1.79, 3.42) p=.53
Brainstem	0.05 (-0.06, 0.17) p=.36	-0.05 (-0.17, 0.06) p=.37	-0.13 (-0.32, 0.05) p=.15	1.45 (-0.92, 3.81) p=.23
Cerebrospinal fluid	-0.06 (-0.21, 0.09) p=.42	-0.12 (-0.26, 0.01) p=.07	-0.12 (-0.32, 0.09) p=.26	0.66 (-1.75, 3.07) p=.59

Abbreviations: EF = executive function; MRI = magnetic resonance imaging

Supplementary Figure S1. Missingness-directed acyclic graph, depicting the assumptions made regarding the causes of missing data.

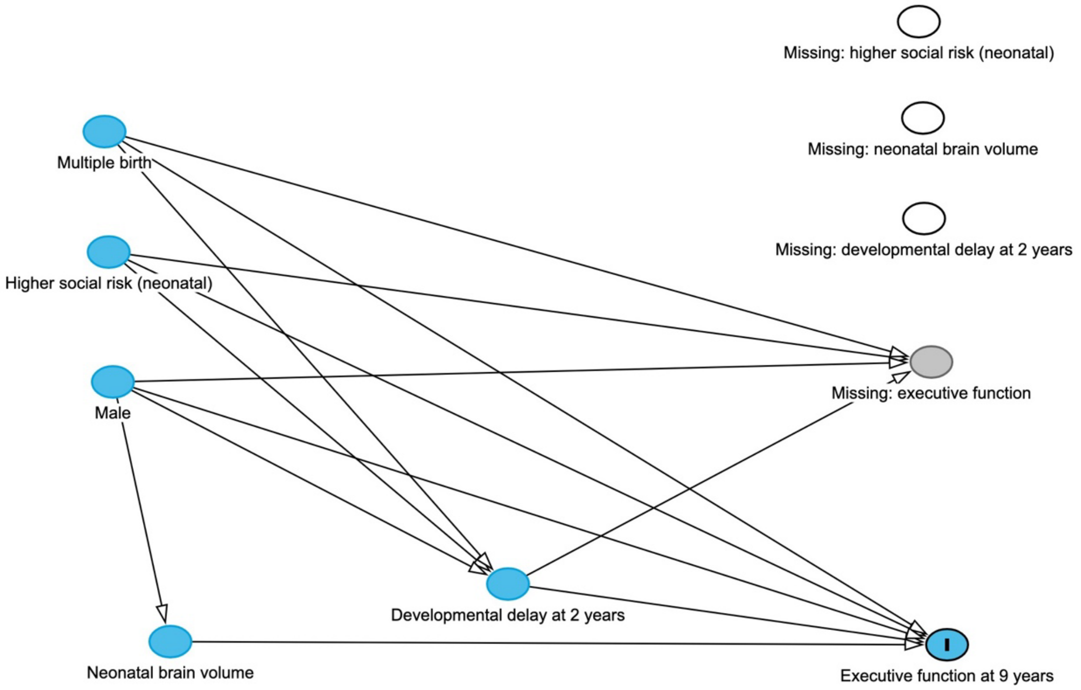

This figure depicts our assumptions about the causes of missingness for executive function data at 9 years. The regression coefficient between neonatal brain volume and executive function outcomes at 9 years can be consistently estimated from the observed data under these assumptions. A complete case analysis would result in selection bias, while a multiple imputation procedure is expected to reduce this bias. The multiple imputation procedure included the executive function outcome at 9 years, the brain volume variable, as well as neonatal social risk, multiple birth, sex and developmental delay at 2 years. Linear regression was used for the imputation of symmetrically distributed variables and predictive mean matching was used for skewed variables. Logistic regression was used to impute dichotomous variables.
